# Association of chronic inflammation with cardiovascular risk in chronic obstructive pulmonary disease—A cross‐sectional study

**DOI:** 10.1002/hsr2.586

**Published:** 2022-04-07

**Authors:** Đivo Ljubičić, Vedran Balta, Dario Dilber, Hrvoje Vražić, Domagoj Đikić, Dyana Odeh, Jasna Čerkez Habek, Emilija Lozo Vukovac, Neven Tudorić

**Affiliations:** ^1^ Department of Pulmonology Dubrava University Hospital Zagreb Croatia; ^2^ Faculty of Medicine University of Zagreb Zagreb Croatia; ^3^ Faculty of Science University of Zagreb Zagreb Croatia; ^4^ Magdalena Clinic for Cardiovascular Diseases Krapinske Toplice Croatia; ^5^ University North, University Centre Varaždin Varaždin Croatia; ^6^ Clinical Hospital Sveti Duh Zagreb Croatia; ^7^ Department of Pulmonology University Hospital Split Croatia

**Keywords:** arterial stiffness, biological systems inflammatory markers, cardiovascular risk, COPD, lung function

## Abstract

**Background and Aims:**

COPD is progressive lung disease with known higher cardiovascular (CV) risk, mainly attributed to smoking of cigarettes as the main etiological factor of disease. The aim of this study was to compare CV risk in patients with COPD to control groups of smokers and non‐COPD and to investigate the relation of lung function variables, COPD severity, and smoking with Systemic Coronary Risk Estimation (SCORE) risk calculation, arterial stiffness (AS) values, and biological systemic inflammatory markers.

**Methods:**

A total of 208 subjects were included in this study: 61 subjects diagnosed with COPD, 83 smokers without COPD, and 64 nonsmokers without COPD. Medical history and clinical data were recorded, including assessment of pulmonary function and AS, calculation of ankle‐brachial index, blood analysis, and CV risk assessment by SCORE risk calculation.

**Results:**

Subjects with COPD had significantly higher values of SCORE calculation of risk, central aortic pressure, AS, and markers of systemic inflammation compared to control groups of smokers and nonsmokers without COPD (*p* < 0.001). Furthermore, statistically significant increase in hs‐CRP concentration was found between the COPD group and the control group of non‐COPD smokers (*p* < 0.001), and a statistically significantly higher SCORE calculation was found in the COPD group compared to control groups of smokers and nonsmokers without COPD (*p* < 0.001).

**Conclusion:**

The results of the research support further identification and research of biological markers and simple specific tests such as arteriography that will enable progress in personalized treatment of patients with COPD and better primary and secondary prevention of comorbidities with the aim of improved treatment outcome.

## 1 INTRODUCTION

Although COPD is considered to be a lung disease, systemic manifestations of COPD associated with increased morbidity and mortality are increasingly being recognized.^1^ Comorbidities were previously thought to occur only in the later stages of COPD, however, research in recent years indicates a high proportion of patients with comorbidities, even in patients with mild broncho‐obstruction.^2^ Accurately determining the type of comorbidities that occur with COPD regulates treatment choices, occurrence, frequency, and types of patients' future health complications and affect survival rate and time. This is important because today it is known that optimal and early treatment as well as primary and secondary prevention of comorbidities have a clear positive effect on the clinical outcome of COPD. Several prospective studies have described an association between impaired lung function and cardiovascular (CV) morbidity and mortality.^3^, ^4^, ^5^, ^6^ Furthermore, epidemiological data suggest that COPD patients are at higher risk of CV disease compared to control groups by age and sex without COPD.^7^ Also, the systemic inflammatory response is thought to play a possible role in explaining this association.^8^ Besides the already mentioned, patients with mild to moderate COPD activity have been found to die more often from lung cancer and CV disease, such as coronary heart disease, than from the respiratory effects of COPD itself.^9^, ^10^, ^11^

Although it has long been established that inflammation of the small airway mucosa is an initial event in the pathogenesis of COPD and that the severity of inflammation is related to the degree of obstruction, recent studies have indicated that systemic inflammation in COPD may accelerate atherosclerosis.^12^, ^13^ Since atherosclerosis is the result of multiple risk factors, all currently valid guidelines for CV disease prevention in clinical practice recommend an assessment of the overall CV risk. Thus, the Systemic Coronary Risk Estimation (SCORE) chart of fatal CV disease is recommended in the European guidelines for the prevention of CV disease in clinical practice from 2016.^14^, ^15^

Since classical risk factors only indirectly suggest atherosclerotic processes that induce CV changes, the interest of the profession for a simple and noninvasive way of detecting increased CV risk in a subclinical stage at the individual level has increased. SCORE risk assessment is based on classical risk factors (age, smoking, cholesterol, systolic blood pressure [SBP]) and is effective at the population level, but is less accurate for determining specific, individual risk exposure of an individual.^16^ Of the noninvasive methods of CV risk assessment, the method of determining the elasticity of the arterial wall, that is, the assessment of arterial stiffness (AS), has become the most widespread in the last decade, with methods such as pulse wave analysis (PWA), pulse wave velocity (PWV), and aortic augmentation index (AIx) due to their reproducibility and ease of performance.^17^, ^18^ These tests have been shown to be associated with coronary microvascular endothelial function^19^ and aortic PWV as an independent predictor of the CV disease.^20^ Previous research suggests that aortic stiffness has been shown to be an independent predictor of overall and CV morbidity and mortality in hypertensives and healthy subjects in the elderly.^21^, ^22^, ^23^, ^24^ Research by Zureik et al. demonstrates that PWV is significantly and negatively associated with the spirometric parameter of forced expiratory volume in the first second (FEV1).^25^ Furthermore, Sabit et al. compared PWV in COPD patients with healthy smokers and ex‐smokers who did not suffer from the CV disease. PWV was higher in patients with COPD and inversely related to FEV1 values.^26^ A review of the available literature did not find a study that comprehensively investigated the relationships of pulmonary function, CV risk assessment, and AS measured by the oscillometric method as an independent predictor of CV risk in patients with COPD and smokers without COPD and nonsmokers without COPD as control groups.

The main objective of this study was to determine and compare CV risk in patients with COPD to control groups of smokers and nonsmokers without COPD and to investigate the relationship of variables of lung function, severity of COPD, and smoking with SCORE risk calculation results, AS values, and biological markers of systemic inflammation.

## 2 PATIENTS AND METHODS

### 2.1 Patients

The study was conducted as a cross‐section through the population at the Clinical Hospital Dubrava, Zagreb, and patients were included based on a search of the database of the Clinic for Internal Medicine of the Clinical Hospital Dubrava from July 2017 to April 2018.

A total of 208 subjects were included in this study divided into three groups: 61 subjects diagnosed with COPD, 83 smokers without COPD, and 64 nonsmokers without COPD. All subjects in the study, including smokers and nonsmokers without COPD were volunteers and all the diagnostic measures were made in the study time from July 2017 to April 2018.

Criteria for inclusion of subjects with COPD were as follows: men or women between 40 and 70 years of age, confirmed diagnosis of COPD without exacerbation in the last month; nonsmokers‐someone who has not smoked more than 100 cigarettes in their lifetime and does not currently smoke; smokers‐current whose pack‐years index (PYI) ≥ 10 or former—someone who has smoked more than 100 cigarettes in their lifetime, but has not smoked in the last 28 days; patients receiving adequate therapy for COPD and patients without initiating therapy. Criteria for inclusion of non‐COPD smokers were: men or women between 40 and 70 years of age, smokers‐current or former with an intensity index with PYI ≥ 10. Criteria for inclusion of non‐COPD nonsmokers were: men or women between the ages of 40 and 70 years.

The excluded study criteria applied both to cases and controls were: persons suffering from lung diseases (active tuberculosis, bronchiectasis, pneumonia, lung cancer, lung fibrosis), patients on continuous oxygen therapy, inability to perform lung function tests, proven coronary heart disease or atherosclerotic disease of peripheral arteries, tachyarrhythmia or bradyarrhythmia of the heart and clinically manifest heart failure, unregulated diabetes, chronic renal failure, active rheumatic disease, autoimmune disease, and unregulated or poorly regulated arterial hypertension with mean values above 140/90 mmHg, respectively.

Anamnestic data were recorded in all subjects: physical examination was performed, and physical and anthropometric measurements were performed on calibrated devices: body weight, height, arterial pressure measurement with an oscillometric pressure gauge, pulse rate, and hemoglobin oxygen saturation. In the groups of subjects with COPD, the disease was assessed by BODEx index (BMI, FEV1, mMRC scale of dyspnea, frequency of exacerbations of COPD), and the method of pharmacological treatment of COPD was recorded in each subject.^27^

## 3 METHODS

### 3.1 Ethical approval details and informed consent

In conducting the research, the laws of the Republic of Croatia and international conventions were fully respected. The study was approved by the Ethics Committee of the Medical Faculty of the University of Zagreb and the Ethics Committee of the Clinical Hospital Dubrava (No. 380‐59‐10106‐16‐20/269). All subjects were familiar with the conduct of the study and signed an informed consent to participate in the study.

### 3.2 Assessment of pulmonary function

The assessment of pulmonary function was determined and GOLD criterion was applied and applied criterion was assessed postbronchodilator by spirometry according to ISO standards IS9001 and ISO13485 using a Minispir® Light spirometer in accordance with the recommendations of the European Respiratory Society.^28^ The following parameters were measured by spirometry: forced expiratory volume in the first second (FEV1), forced vital capacity (FVC), Tiffeneau–Pinelli index (FEV1/FVC), and airflow through the small airways. Using disposable, factory‐calibrated nozzle airflow sensors, FlowMir®, it was not necessary to calibrate the device. The obtained data were analyzed using the computer program Winspiro PRO® PC.

### 3.3 Arterial blood gas analysis

Blood for gas analysis of arterial blood was obtained by taking blood from the *arteria radialis*.^29^ Blood was analyzed in a Gem premier 3000 analyser, Vetusi. Arterial blood gas analysis was used to analyze partial oxygen pressure (*p*O_2_/kPa), carbon dioxide partial pressure (*p*CO_2_/kPa), pH value, hydrogen carbonate ion concentration (mmol/L), and oxygen saturation (%).

### 3.4 Analysis of hematological and biochemical parameters

Blood for analysis of hematological and biochemical parameters was sampled by venepuncture of the cubital vein using Vacutainer® tubes with K_3_EDTA with anticoagulant (hematology) and without anticoagulant (biochemistry). Complete blood count (CBC) was determined in a Siemens Advia 2120i hematology analyser (Siemens Healthcare Diagnostics). For the assessment of biochemical parameters, the serum was isolated by centrifuging the blood at 1300 rpm for 10 min using a 32 Rotofix A centrifuge (Andreas Hettich GmbH & Co). From the biochemical parameters, the levels of glucose, urea, creatinine, triglycerides, total cholesterol, HDL‐cholesterol, LDL‐cholesterol, and hs‐CRP were determined. These parameters were analyzed in Beckman Coulter AU2700 plus and AU680 biochemical analysers. Serum hs‐CRP concentration was determined by a highly specific immunoturbidimetric method on latex particles,^30^ while fibrinogen concentration was determined by a modified Clauss coagulometric method^31^ using BCS XP device (Siemens Healthcare Diagnostics, USApo). In accordance with the standard ISO norms HR EN ISO 15189, according to which the Clinical Department for Laboratory Diagnostics of the Clinical Hospital Dubrava is accredited, the accuracy of the results is guaranteed by calibrating test and releasing a control sample of known concentration declared by the manufacturer.

### 3.5 Assessment of AS

AS was determined by measuring the aortic pulse wave velocity (PWVAo) and the AIX using a noninvasive TensioMed Arteriograph device and associated software (TensioMed Software v.1.10.0.2, TensioMed). The distance from the aortic arch to the iliac bifurcation was approximated by measuring the distance between the sternal jugulum and the pubic symphysis.^32^ PWVAо and AIX values were presented as the mean values of the two measurements. The standard deviation (SD) was calculated for each beat when measured for 8 s.

### 3.6 Ankle‐brachial index (ABI)

The ABI is the ratio of the SBP measured at the ankle to that measured at the brachial artery of the arm.^33^ The blood pressure cuff is inflated proximal to the brachial artery of the arm and foot's posterior tibial or dorsalis pedis artery and systolic pressure on the foot determined by the Doppler *Ultrasound* with 8 megahertz peripheral probe. The highest ABPI ratio An ABPI between and including 0.90 and 1.29 considered normal, while a lesser than 0.9 indicates arterial disease, and an ABPI value of 1.3 or greater suggests severe calcification of the walls.

### 3.7 CV risk assessment by SCORE risk calculation

In addition to the above methods, the assessment of increased CV risk was determined using the SCORE table of calculations for high‐risk countries according to the guidelines for the prevention of CV disease in clinical practice from 2016.^15^

### 3.8 Statistical analyses

Before conducting the research and planning the size of individual groups of subjects, a test power analysis for one‐way analysis of variance (ANOVA) was previously performed with the following parameters: significance level *α* = 0.05, effect size *f* = 0.25, and three study groups. The minimum required total sample should have been 159 respondents or 53 per group. Given the possible deviations in the variability of the examined differences, a minimum number of subjects of 60 per group (180 in total) was predicted as a safety factor, which gives a satisfactory test power of 85%. Test power analysis was performed using G* Power for Windows version 3.1.2.

IBM SPSS Statistics software, version 23 was used to process the results obtained after conducting the research in statistical analysis. Quantitative values were analyzed by Kolmogorov–Smirnov test and in the further analysis appropriate parametric statistical tests and data display methods were applied. Quantitative values are presented as mean and SD and 95% confidence intervals (95% CIs), while categorical values are presented in absolute numbers and corresponding proportions. One‐way ANOVA was performed to establish significant differences between all three study groups (COPD, smokers without COPD, and nonsmokers without COPD). After the analysis of variance, a post hoc analysis according to Bonferroni with age and sex adjustment was additionally performed, to show the significance of individual interrelationships between each of the examined groups. The association of pulmonary parameters with CV risks was determined by Pearson's correlation coefficients (*r*), where the absolute value of the correlation coefficient >0.600 was rated as a strong correlation, from 0.300 to 0.599 as a moderate correlation, and <0.300 as a weak correlation. All *p* values less than 0.05 were considered significant. Spearman's correlation coefficients rho (*r*_s_) were used in the calculation of correlations of COPD phenotype and COPD diagnosis with CV risk parameters, given the nonparametric distribution of COPD phenotype and diagnosis according to GOLD.

## 4 RESULTS

### 4.1 Socio‐demographic and clinical characteristics of the examinees

According to the analysis of the socio‐demographic and clinical characteristics of the respondents (Table 1), over two‐thirds of respondents (69.2%) in the nonsmoking group were female, while in the smoking group there were 51 (61.4%) women and in the COPD group only 20 (32.8%) female (*p* < 0.001). In the COPD group, the majority of the respondents were between 55 and 64 years of age (54.1%), while in the groups of smokers and non‐smokers without COPD symptoms, the age group was younger than 55 years. In the COPD group, 43 subjects (70.5%) actively smoked, and this prevalence was statistically significantly different from the group of smokers who did not have COPD symptoms (67.5%, *p* = 0.019). Within the COPD group, the level of obstruction according to GOLD type 2: 23 prevails (37.7%). Most subjects with COPD were assigned to Groups A, B, and D, while the fewest were in Group C (Table 2). The most common phenotype in the study population was a nonexacerbator (72.1%), and the least was an exacerbator with chronic bronchitis (6.6%) and subjects with asthma‐overlap syndrome and COPD (3.3%). 21.3% of subjects were not included in pharmacological treatment, which also coincides with the percentage of subjects in the GOLD A group (21.3%). Shortness of breath was rated by grade 50.0% of respondents according to the mMRC scale, while over 50% of them did not have an exacerbation of the disease in the previous year. Compared to active smokers without COPD symptoms, smokers with COPD symptoms had a statistically significantly longer smoking history (*p* < 0.001), smoked on average almost seven cigarettes more (*p* = 0.001), and had twice the PYI (*p* < 0.001). The analysis of anthropometric variables (height, body weight, BMI, jugulum‐symphysis distance) did not reveal statistically significant differences between the examined groups.

**Table 1:** Socio‐demographic and clinical characteristics of the examinees

		Groups					
		COPD (*N* = 61)		Smokers without COPD (*N* = 83)		Nonsmokers without COPD (*N* = 65)	
		*N*	%	*N*	%	*N*	%
Gender	Male	41	67.2	32	38.6	20	30.8
	Female	20	32.8	51	61.4	45	69.2
Age	<55 years	8	13.1	55	66.3	46	70.8
	55–64 years	33	54.1	23	27.7	15	23.1
	≥65 years	20	32.8	5	6.0	4	6.2
Smoking	Nonsmoker	5	8.2	0	0.0	65	100.0
	Smoker	43	70.5	56	67.5	0	0.0
	Ex‐smoker	13	21.3	27	32.5	0	0.0
Presence of COPD	No	0	0.0	83	100.0	65	100.0
	Yes	61	100.0	0	0.0	0	0.0
GOLD obstruction	0	0	0.0	83	100.0	65	100.0
	1	11	18.0	0	0.0	0	0.0
	2	23	37.7	0	0.0	0	0.0
	3	18	29.5	0	0.0	0	0.0
	4	9	14.8	0	0.0	0	0.0

**Table 2:** Clinical characteristics of the group diagnosed with COPD (*N* = 61)

		*N*	%
COPD diagnosis according to GOLD	A	13	21.3
	B	21	34.4
	C	5	8.2
	D	22	36.1
COPD phenotype	Nonexacerbator (<2 years)—chronic bronchitis or emphysema	44	72.1
	Exacerbator with chronic bronchitis	4	6.6
	Exacerbator with emphysema	11	18.0
	ACOS	2	3.3
COPD therapy	No	13	21.3
	Yes	48	78.7
mMRC	0	7	11.7
	1	6	10.0
	2	30	50.0
	3	16	26.7
	4	1	1.7
Frequency of COPD exacerbations	0	31	50.8
	1	21	34.4
	2	9	14.8

Abbreviations: ACOS, asthma‐COPD overlap syndrome; mMRC, modified Medical Research Council.

### 4.2 Differences between examined groups

By analyzing the results of spirometric parameters (Table 3), it was found that subjects in the COPD group had statistically significantly lower values of all spirometric parameters compared to control groups of smokers and nonsmokers without COPD (*p* < 0.001), while there were no statistically significant differences observed between smokers and nonsmokers.

**Table 3:** Differences of spirometric parameters between examined groups

Parameters	Groups	*N*	Mean ± SD	95% CI		Min	Max
				Lower bound	Upper bound		
FEV_1_(L)	COPD	61	1.65 ± 0.8^a^	1.44	1.86	0.20	3.93
	Smokers without COPD	83	3.15 ± 0.79^a^	2.98	3.32	1.87	5.33
	Nonsmokers without COPD	64	3.13 ± 0.72^a^	2.95	3.31	1.89	4.93
FEV_1_(%)	COPD	61	55.66 ± 23.87^a^	49.55	61.78	11.00	111.00
	Smokers without COPD	83	98.09 ± 16.06^a^	94.58	101.59	3.30	129.00
	Nonsmokers without COPD	64	102.22 ± 12.68^a^	99.05	105.39	68.00	133.00
FVC (L)	COPD	61	3.22 ± 1.06^a^	2.95	3.49	0.51	6.37
	Smokers without COPD	83	4.00 ± 1.02^a^	3.78	4.23	2.32	6.72
	Nonsmokers without COPD	64	3.94 ± 0.95^a^	3.70	4.18	2.30	6.42
FVC (%)	COPD	61	89.34 ± 27.74^a^	82.24	96.44	18.00	194.00
	Smokers without COPD	83	103.75 ± 16.99^a^	100.04	107.46	3.98	132.00
	Nonsmokers without COPD	64	108.06 ± 13.72^a^	104.64	111.49	64.00	148.00
FEV_1_/FVC (%)	COPD	61	49.84 ± 14.68^a^	46.08	53.60	0.24	75.00
	Smokers without COPD	83	78.91 ± 77.85^a^	77.85	79.97	66.10	89.70
	Nonsmokers without COPD	64	79.56 ± 4.22^a^	78.51	80.62	67.70	88.40
MEF_25‐75_(%)	COPD	58	25.94 ± 15.60^a^	21.84	30.04	5.00	66.00
	Smokers without COPD	83	81.87 ± 20.95^a^	77.30	86.45	3.56	133.00
	Nonsmokers without COPD	64	84.78 ± 17.08^a^	80.52	89.05	46.00	143.00

Abbreviations: CI, confidence interval; FEV_1_, forced expiratory volume in 1 s; FEV_1_/FVC, Tiffeneau–Pinelli index; FVC, forced vital capacity; Max, maximum; MEF_25–75_, maximum expiratory flow in the middle half of the forced expiratory maneuver; Min, minimum; SD, standard deviation.

^a^ The values marked with different superscript letters are significantly different (*p* < 0.05).

Furthermore, analyses of the results of blood pressure, AS and ejection fraction parameters in the COPD group indicate statistically significantly increased values of SBP, mean arterial pressure, PWVAо and SBPAо compared to both control groups (*p* < 0.001), while LVET‐ and *P*‐wave return times indicate statistically significantly less (*p* < 0.001) (Table 4). The average SD in all PWVAo measurements using the Arteriograph was below 1.1 m/s, which indicates excellent measurement quality. Also, the COPD group indicated statistically significantly increased values of pulse pressure and brachial pressure compared to the control group of smokers without COPD and diastolic pressure and heart rate per minute compared to the control group of nonsmokers without COPD (*p* < 0.05). No statistically significant differences were observed between the control groups of smokers and nonsmokers without COPD.

**Table 4:** Differences in blood pressure parameters, arterial stiffness, and ejection fraction between the examined groups

Parameters	Groups	*N*	Mean ± SD	95% CI		Min	Max
				Lower bound	Upper bound		
Systolic BP (mmHg)	COPD	61	139.57 ± 14.23^a^	135.93	143.22	107.00	177.00
	Smokers without COPD	83	130.87 ± 13.94^a^	127.82	133.91	106.00	195.00
	Nonsmokers without COPD	64	130.36 ± 14.76^a^	126.67	134.05	102.00	169.00
Diastolic BP (mmHg)	COPD	61	84.52 ± 9.06^a^	82.20	86.85	67.00	100.00
	Smokers without COPD	83	81.43 ± 8.84^a^	79.50	83.36	60.00	102.00
	Nonsmokers without COPD	64	77.94 ± 9.56^a^	75.55	80.33	56.00	99.00
Heart rate (beats/min)	COPD	61	75.61 ± 12.02^a^	72.53	78.68	51.00	110.00
	Smokers without COPD	83	75.16 ± 11.72^a^	72.60	77.72	53.00	128.00
	Nonsmokers without COPD	64	70.56 ± 9.93^a^	68.08	73.04	52.00	91.00
Mean arterial pressure (mmHg)	COPD	61	102.93 ± 9.91^a^	100.40	105.47	80.00	124.00
	Smokers without COPD	83	97.84 ± 10.02^a^	95.66	100.03	75.00	133.00
	Nonsmokers without COPD	64	95.36 ± 10.44^a^	92.75	97.97	72.00	120.00
Pulse pressure (mmHg)	COPD	61	55.38 ± 10.57^a^	52.67	58.08	34.00	79.00
	Smokers without COPD	83	49.57 ± 9.08^a^	47.58	51.55	33.00	93.00
	Nonsmokers without COPD	64	52.48 ± 10.42^a^	49.88	55.09	36.00	85.00
Brachial AIx (%)	COPD	61	−7.80 ± 28.30^a^	−15.05	−0.55	‐64.60	55.10
	Smokers without COPD	83	−21.55 ± 27.28^a^	−27.50	−15.59	‐81.20	41.30
	Nonsmokers without COPD	63	−16.77 ± 28.25^a^	−23.89	−9.66	‐65.40	37.50
Aortic AIx (%)	COPD	61	33.69 ± 14.33	30.02	37.36	4.90	65.50
	Smokers without COPD	83	26.73 ± 13.80	23.71	29.74	‐3.50	58.50
	Nonsmokers without COPD	63	28.59 ± 13.95	25.07	32.10	4.50	56.60
LVET (ms)	COPD	61	281.07 ± 34.36^a^	272.26	289.87	160.00	345.00
	Smokers without COPD	83	296.63 ± 25.48^a^	291.06	302.19	190.00	360.00
	Nonsmokers without COPD	63	307.14 ± 23.79^a^	301.15	313.13	250.00	355.00
RT (ms)	COPD	61	92.16 ± 19.42^a^	87.19	97.14	50.00	154.00
	Smokers without COPD	83	111.24 ± 17.72^a^	107.37	115.11	65.00	152.00
	Nonsmokers without COPD	63	116.71 ± 20.45^a^	111.56	121.86	75.00	177.00
PWVAo (m/s)	COPD	61	11.67 ± 2.72^a^	10.97	12.36	7.50	19.60
	Smokers without COPD	83	9.58 ± 1.61^a^	9.23	9.93	6.90	14.80
	Nonsmokers without COPD	63	9.09 ± 1.61^a^	8.68	9.49	6.00	13.80
SD_PWVAo_(m/s)	COPD	38	0.95 ± 0.62^a^	0.75	1.16	0.00	2.20
	Smokers without COPD	51	0.59 ± 0.35^a^	0.50	0.69	0.00	1.70
	Nonsmokers without COPD	40	0.57 ± 0.50^a^	0.41	0.73	0.00	2.60
SBPAo (mmHg)	COPD	61	138.92 ± 16.61^a^	134.67	143.17	99.60	185.80
	Smokers without COPD	83	125.25 ± 18.60^a^	121.19	129.31	11.90	175.70
	Nonsmokers without COPD	63	126.74 ± 17.59^a^	122.31	131.17	92.00	177.40
PPAo (mmHg)	COPD	61	53.23 ± 14.67	49.47	56.99	7.00	87.80
	Smokers without COPD	83	45.14 ± 9.12	43.15	47.13	29.10	73.70
	Nonsmokers without COPD	63	55.05 ± 45.45	43.60	66.50	28.70	393.00

Abbreviations: AIx, augmentation index; BP, blood pressure; CI, confidence interval; LVET, left ventricular ejection time; Min, minimum; Max, maximum; PPAo, aortic pulse pressure; PWVAo, aortic pulse wave velocity; RT, return time; SBPAo, aortic systolic blood pressure; SD, standard deviation; SDPWVAo, standard deviation of PWVAo.

^a^ The values marked with different superscript letters are significantly different (*p* < 0.05).

By analyzing the biochemical parameters (Table 5), the statistically most significant changes were visible in the decrease in hemoglobin oxygen saturation and the increased concentration of fibrinogen in the COPD group compared to both control groups (*p* < 0.001). Also, a statistically significant increase in hs‐CRP concentration was found between the COPD group and the control group of non‐COPD smokers (*p* < 0.001), while statistically significant differences in serum triglyceride concentrations were found only between control groups of smokers and nonsmokers without COPD (*p* < 0.05).

**Table 5:** Differences in biochemical parameters between the examined groups

Parameters	Groups	*N*	Mean ± SD	95% CI		Min	Max
				Lower bound	Upper bound		
Oxygen saturation (%)	COPD	61	90.77 ± 17.51^a^	86.28	95.25	94.00	98.00
	Smokers without COPD	83	97.57 ± 1.28^a^	97.29	97.85	94.00	100.00
	Nonsmokers without COPD	65	97.55 ± 1.30^a^	97.23	97.88	94.00	100.00
Glucose (mmol/L)	COPD	60	5.68 ± 1.23	5.36	6.00	2.40	9.40
	Smokers without COPD	81	5.77 ± 2.63	5.18	6.35	2.60	25.40
	Nonsmokers without COPD	65	5.04 ± 0.96	4.81	5.28	2.20	7.60
Urea (mmol/L)	COPD	60	5.61 ± 1.85	5.13	6.08	2.10	10.30
	Smokers without COPD	81	5.37 ± 1.80	4.98	5.77	2.20	13.00
	Nonsmokers without COPD	65	5.46 ± 1.45	5.11	5.82	2.30	9.80
Creatinine (µmol/L)	COPD	60	81.08 ± 18.39	76.33	85.83	41.00	161.00
	Smokers without COPD	81	81.95 ± 16.42	78.32	85.58	35.00	136.00
	Nonsmokers without COPD	63	84.49 ± 19.25	79.64	89.34	32.00	124.00
Triglycerides(mmol/L)	COPD	60	2.01 ± 1.25^a^	1.68	2.33	0.70	8.60
	Smokers without COPD	81	2.62 ± 1.98^a^	2.18	3.05	0.60	13.50
	Nonsmokers without COPD	65	1.86 ± 1.12^a^	1.58	2.14	0.70	7.10
Total cholesterol (mmol/L)	COPD	61	5.56 ± 1.20	5.25	5.87	2.60	8.60
	Smokers without COPD	81	5.41 ± 1.21	5.15	5.68	1.50	9.10
	Nonsmokers without COPD	65	5.40 ± 1.35	5.06	5.73	1.90	9.10
HDL‐cholesterol (mmol/L)	COPD	60	1.42 ± 0.36	1.33	1.51	0.70	2.20
	Smokers without COPD	81	1.41 ± 0.33	1.34	1.49	0.50	2.50
	Nonsmokers without COPD	65	1.44 ± 0.33	1.36	1.52	0.70	2.20
LDL‐cholesterol (mmol/L)	COPD	59	3.58 ± 0.97	3.33	3.83	1.60	5.80
	Smokers without COPD	81	3.26 ± 0.98	3.05	3.48	0.80	6.20
	Nonsmokers without COPD	64	3.32 ± 1.12	3.04	3.60	0.80	6.30
hs‐CRP (g/L)	COPD	61	6.81 ± 12.74^a^	3.55	10.08	0.10	64.90
	Smokers without COPD	81	1.61 ± 1.42^a^	1.30	1.93	0.10	6.90
	Nonsmokers without COPD	64	3.56 ± 8.42^a^	1.46	5.66	0.10	45.40
Fibrinogen (g/L)	COPD	57	3.93 ± 1.24^a^	3.60	4.26	1.20	9.20
	Smokers without COPD	82	3.18 ± 0.97^a^	2.97	3.39	0.30	8.00
	Nonsmokers without COPD	57	3.36 ± 0.83^a^	3.14	3.58	1.00	5.80

Abbreviations: CI, confidence interval; HDL, high‐density lipoprotein; hs‐CRP, high sensitivity protein; LDL, low‐density lipoprotein; Max, maximum; Min, minimum; SD, standard deviation.

^a^ The values marked with different superscript letters are significantly different (*p* < 0.05).

The analysis of hematological parameters (Table 6) revealed statistically significantly increased values of erythrocytes, hemoglobin, MCH, and neutrophils in the COPD group compared to the control group of nonsmokers without COPD (*p* < 0.05), while the hematocrit value was statistically significantly increased compared to both control groups (*p* < 0.001). Also, a statistically significantly lower number of leukocytes was found in the control group of nonsmokers without COPD compared to the group of COPD and the control group of smokers (*p* < 0.05), while the number of lymphocytes was statistically significantly reduced in the group of COPD compared to the group of smokers without COPD. a (*p* < 0.05).

**Table 6:** Differences in hematological parameters between the examined groups

Parameters	Groups	*N*	Mean ± SD	95% CI		Min	Max
				Lower bound	Upper bound		
Leukocytes (×10^9^/L)	COPD	60	7.92 ± 2.04^a^	7.39	8.45	3.80	13.10
	Smokers without COPD	82	7.93 ± 2.01^a^	7.49	8.37	4.70	14.00
	Nonsmokers without COPD	64	7.05 ± 1.85^a^	6.59	7.52	2.60	11.70
Erythrocytes (×10^12^/L)	COPD	60	4.78 ± 0.46^a^	4.66	4.90	3.69	6.28
	Smokers without COPD	81	4.62 ± 0.52^a^	4.50	4.73	3.62	6.73

	Nonsmokers without COPD	63	4.54 ± 0.42^a^	4.44	4.65	3.89	5.70
Hemoglobin (g/L)	COPD	60	142.34 ± 21.79^a^	136.71	147.97	1.38	173.00
	Smokers without COPD	82	137.55 ± 14.90^a^	134.27	140.82	97.00	175.00
	Nonsmokers without COPD	64	133.80 ± 13.72^a^	130.37	137.22	91.00	158.00
Hematocrit (L/L)	COPD	60	0.44 ± 0.03^a^	0.43	0.45	0.36	0.53
	Smokers without COPD	81	0.42 ± 0.05^a^	0.41	0.43	0.29	0.54
	Nonsmokers without COPD	63	0.40 ± 0.04^a^	0.40	0.41	0.29	0.49
MCV (fl)	COPD	60	90.47 ± 11.64	87.46	93.48	9.30	104.90
	Smokers without COPD	81	90.70 ± 4.22	89.77	91.63	73.90	97.20
	Nonsmokers without COPD	63	89.19 ± 4.86	87.97	90.41	74.10	103.60
MCH (pg)	COPD	60	30.32 ± 1.53^a^	29.93	30.72	27.20	33.60
	Smokers without COPD	81	29.81 ± 1.70^a^	29.44	30.19	23.60	32.90
	Nonsmokers without COPD	63	29.49 ± 1.84^a^	29.03	29.96	23.10	33.90
MCHC (g/L)	COPD	60	330.30 ± 6.55	328.61	331.99	315.00	345.00
	Smokers without COPD	81	328.49 ± 7.60	326.81	330.17	302.00	348.00
	Nonsmokers without COPD	63	330.60 ± 7.83	328.63	332.57	311.00	347.00
RDW (%)	COPD	60	13.84 ± 0.89	13.61	14.07	12.60	17.70
	Smokers without COPD	81	13.68 ± 1.32	13.39	13.97	11.80	20.50
	Nonsmokers without COPD	63	13.53 ± 0.87	13.31	13.75	12.30	18.20
Thrombocytes (×10^9^/L)	COPD	60	246.03 ± 63.54	229.62	262.45	133.00	455.00
	Smokers without COPD	82	240.96 ± 51.28	229.70	252.23	145.00	357.00
	Nonsmokers without COPD	64	231.36 ± 52.30	218.29	244.42	123.00	374.00
MPV (fl)	COPD	60	8.32 ± 0.9	8.08	8.56	6.90	12.60
	Smokers without COPD	81	9.72 ± 9.4	7.64	11.80	7.20	93.00
	Nonsmokers without COPD	63	8.48 ± 0.9	8.25	8.71	7.10	11.30
Neutrophils (×10^9^/L)	COPD	60	5.05 ± 1.80^a^	4.58	5.51	2.10	9.90
	Smokers without COPD	81	4.78 ± 1.55^a^	4.44	5.12	2.50	10.10
	Nonsmokers without COPD	64	4.14 ± 1.59^a^	3.75	4.54	0.90	9.90
Lymphocytes (×10^9^/L)	COPD	60	2.00 ± 0.61^a^	1.84	2.16	0.80	3.30
	Smokers without COPD	81	2.37 ± 0.67^a^	2.22	2.51	1.10	4.60
	Nonsmokers without COPD	64	2.17 ± 0.59^a^	2.02	2.31	1.00	3.70
Monocytes (×10^9^/L)	COPD	60	0.55 ± 0.16	0.50	0.59	0.20	1.00
	Smokers without COPD	81	0.54 ± 0.20	0.50	0.58	0.20	1.20
	Nonsmokers without COPD	64	0.49 ± 0.18	0.44	0.54	0.20	1.00
Eosinophils (×10^9^/L)	COPD	60	0.17 ± 0.17	0.13	0.22	0.00	1.20
	Smokers without COPD	81	0.16 ± 0.12	0.13	0.18	0.00	0.70
	Nonsmokers without COPD	64	0.14 ± 0.11	0.11	0.16	0.00	0.60
Basophils (×10^9^/L)	COPD	60	0.05 ± 0.05	0.03	0.06	0.00	0.10
	Smokers without COPD	81	0.03 ± 0.05	0.02	0.05	0.00	0.20
	Nonsmokers without COPD	64	0.03 ± 0.07	0.01	0.05	0.00	0.40
LUC (×10^9^/L)	COPD	45	0.13 ± 0.05	0.12	0.15	0.10	0.30
	Smokers without COPD	33	0.15 ± 0.06	0.13	0.17	0.10	0.30
	Nonsmokers without COPD	36	0.15 ± 0.08	0.12	0.18	0.10	0.40

Abbreviations: CI, confidence interval; LUC, large unstained cell; Max, maximum; MCH, mean corpuscular hemoglobin; MCHC, mean corpuscular hemoglobin concentration; MCV, mean corpuscular volume; Min, minimum; MPV, mean platelet volume; RDW, red cell distribution width; SD, standard deviation.

^a^ The values marked with different superscript letters are significantly different (*p* < 0.05).

The results of the analysis of heart risk parameters indicate a statistically significantly higher SCORE calculation of heart risk in the COPD group compared to control groups of smokers and nonsmokers without COPD (*p* < 0.001), while statistically significant differences with respect to ABI index values were found only between control group of smokers and nonsmokers without COPD (*p* < 0.05) (Table 7).

**Table 7:** Differences in heart risk parameters and ABI index between the examined groups

Parameters	Groups	*N*	Mean ± SD	95% CI		Min	Max
				Lower bound	Upper bound		
SCORE risk calculation	COPD	61	8.49 ± 5.76^a^	7.02	9.97	0.00	26.00
	Smokers without COPD	82	3.01 ± 4.16^a^	2.10	3.93	0.00	22.00
	Nonsmokers without COPD	64	1.88 ± 2.68^a^	1.21	2.54	0.00	13.00
Ankle systolic pressure (mmHg)	COPD	61	133.77 ± 17.3	129.34	138.20	80.00	180.00
	Smokers without COPD	83	133.81 ± 14.88	130.56	137.06	90.00	170.00
	Nonsmokers without COPD	61	130.33 ± 16.53	126.09	134.56	90.00	170.00
ABI	COPD	61	0.96 ± 0.12^a^	0.93	1.00	0.62	1.27
	Smokers without COPD	83	1.03 ± 0.10^a^	1.01	1.05	0.76	1.27
	Nonsmokers without COPD	64	0.96 ± 0.25^a^	0.90	1.02	0.00	1.27

Abbreviations: ABI, ankle‐brachial index; CI, confidence interval; Max, maximum; Min, minimum; SD, standard deviation.

^a^ The values marked with different superscript letters are significantly different (*p* < 0.05).

**Figure 1:**
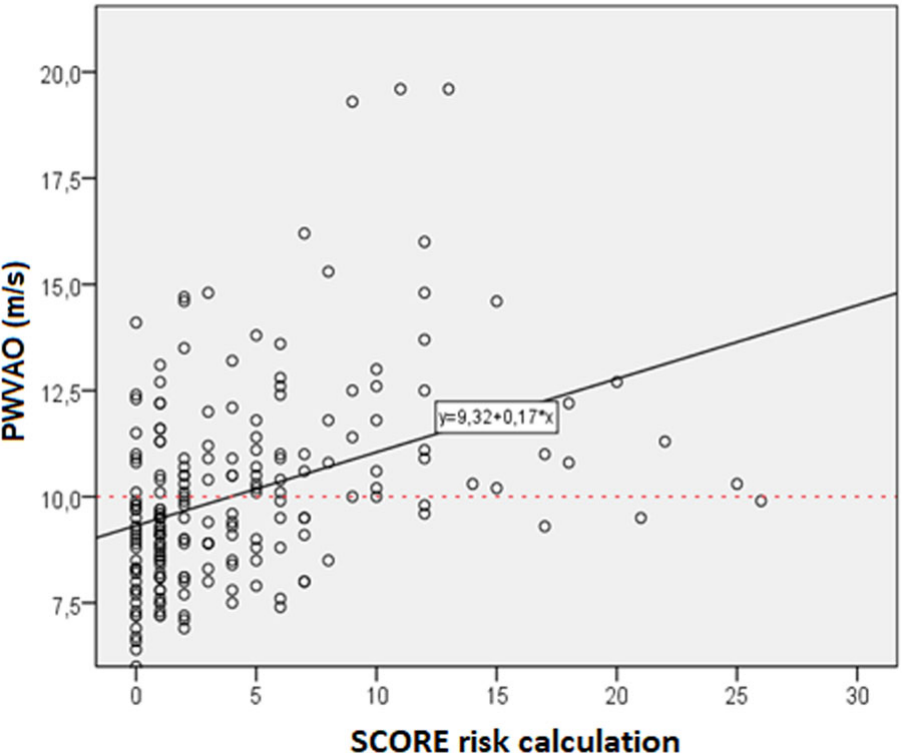
Regression line of significant correlation between PWVAо (m/s) and Systemic Coronary Risk Estimation (SCORE) risk calculation: Pearson's correlation coefficient, *r* = 0.390; *p* < 0.001

**Figure 2:**
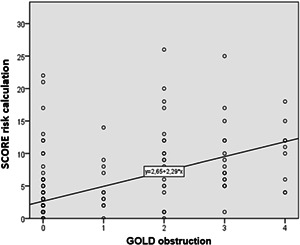
Regression line of significant correlation of obstruction by GOLD and Systemic Coronary Risk Estimation (SCORE) risk calculation: Pearson's correlation coefficient, *r* = 0.542; *p* < 0.001

**Figure 3:**
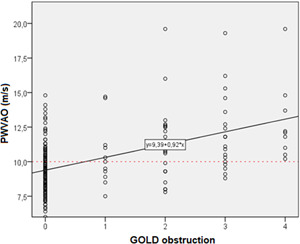
Regression line of significant obstruction correlation by GOLD and PWVAо (m/s): Pearson's correlation coefficient, *r* = 0.496; *p* < 0.001

The level of obstruction according to GOLD was significantly associated with almost all the parameters of AS, and mostly with the values of PWVAo (*r* = 0.496, *p* < 0.001), P2 wave return time (*r* = −0.484, *p* < 0.001), LVET (*r* = −0.331, *p* < 0.001), *p*CO_2_ (*r* = 0.522, *p* < 0.001), *p*O_2_ (*r* = −0.615, *p* < 0.001), SBP‐Ao (*r* = 0.294, *p* < 0.001) and SCORE by risk calculation 0.542, *p* < 0.001) (Tables 8 and 9). These results suggest that a higher level of obstruction is associated with higher AS values, decreased O_2_ concentration, increased CO_2_ concentration, and higher SCORE calculation of CV risk (Figures 1, 2, 3).

**Table 8:** Correlation coefficients of pulmonary function and smoking status parameters with values of body mass index, blood pressure, and arterial stiffness parameters

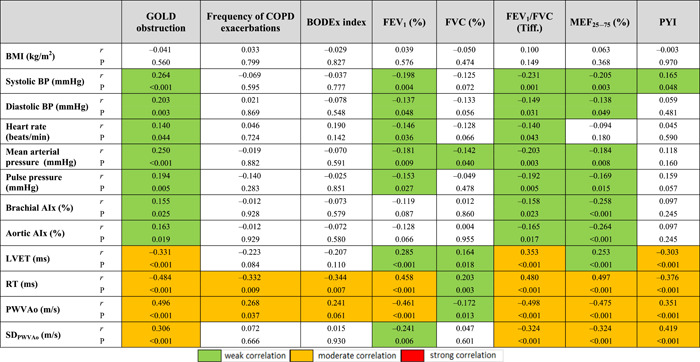

Abbreviations: AIx, augmentation indeks; BMI, body mass indeks; BP, blood pressure; BODEX, body mass indeks, airflow obstruction, dyspnea, and exacerbations indeks; FEV_1_, forced expiratory volume in 1 s; FEV_1_/FVC, Tiffeneau–Pinelli index; FVC, forced vital capacity; LVET, left ventricular ejection time; MEF_25__–__75_, maximum expiratory flow in the middle half of the forced expiratory maneuver; PWVAo, aortic pulse wave velocity; PYI, pack‐years index; *r*, correlation coefficient; RT, return time; SD_PWVAo_, standard deviation of PWVAo.

**Table 9:** Correlation coefficients of pulmonary function and smoking status parameters with values of central blood pressure, pH, *p*CO_2_, *p*O_2_, ABI, and SCORE risk calculation

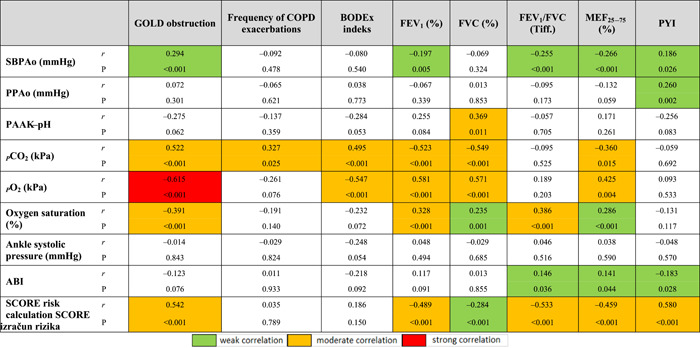

Abbreviations: ABI, ankle‐brachial index; BODEX, body mass index, airflow obstruction, dyspnea, and exacerbations index; FEV1, forced expiratory volume in 1 s; FEV_1_/FVC, Tiffeneau–Pinelli index; FVC, forced vital capacity; MEF_25–75_, maximum expiratory flow in the middle half of the forced expiratory maneuver; SBPAo, aortic systolic blood pressure; PPAo, aortic pulse pressure; PYI, pack‐years index; *r*, correlation coefficient.

A significant correlation between the parameters of pulmonary function and the level of obstruction according to GOLD was also found with the measured markers of systemic inflammation (Table 10). Higher levels of obstruction were associated with higher concentrations of hs‐CRP (*r* = 0.243, *p* < 0.001) and fibrinogen (*r* = 0.290, *p* < 0.001). Also, the value of fibrinogen concentration was more strongly associated with the values of FEV1 (*r* = −0.302, *p* < 0.001), FEV1/FVC (*r* = −0.308, *p* < 0.001) and MEF25‐75 (*r* = −0.321, *p* < 0.001).

**Table 10:** Correlation coefficients of pulmonary function parameters and smoking status with biochemical parameters

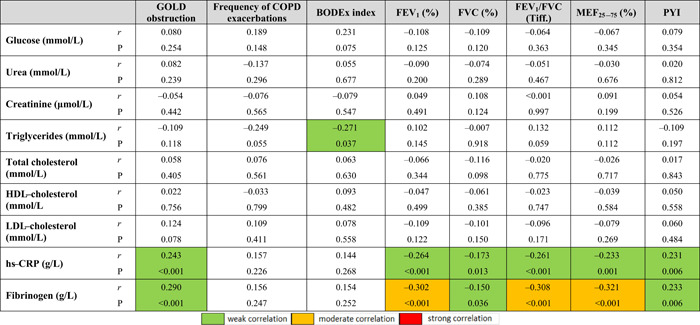

Abbreviations: BODEX, body mass indeks, airflow obstruction, dyspnea, and exacerbations indeks; FEV_1_, forced expiratory volume in 1 s; FVC, forced vital capacity; FEV_1_/FVC, Tiffeneau–Pinelli index; HDL, high‐density lipoprotein; hs‐CRP, high sensitivity protein; LDL, low‐density lipoprotein; MEF_25__–__75_, maximum expiratory flow in the middle half of the forced expiratory maneuver; PYI, pack‐years index; *r*, correlation coefficient.

A significant positive correlation was found between the level of GOLD obstruction with the values of hemoglobin (*r* = 0.224, *p* = 0.001), hematocrit (*r* = 0.296, *p* < 0.001), erythrocytes (*r* = 0.194, *p* = 0.006), and neutrophils (*p* = 0.169, *p* = 0.015), while the association was negative with lymphocyte count (*r* = −0.018, *p* = 0.002) (Table 11).

**Table 11:** Correlation coefficients of pulmonary function parameters and smoking status with hematological parameters

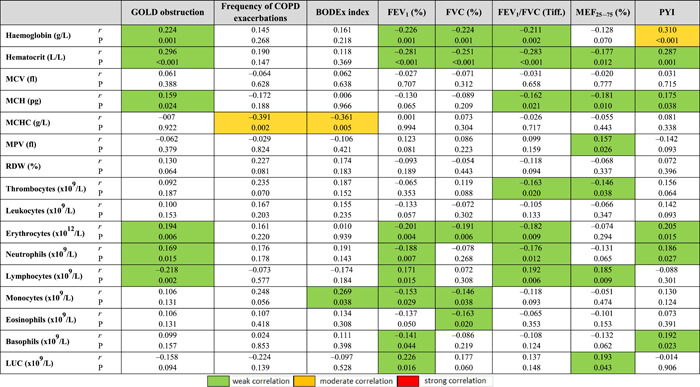

Abbreviations: FEV_1_, forced expiratory volume in 1 s; FVC, forced vital capacity; FEV_1_/FVC, Tiffeneau–Pinelli index; LUC, large unstained cell; MCH, mean corpuscular hemoglobin; MCHC, mean corpuscular hemoglobin concentration; MCV, mean corpuscular volume; MEF_25__–__75_, maximum expiratory flow in the middle half of the forced expiratory maneuver; PYI, pack‐years index; *r*, correlation coefficient; RDW, red cell distribution width; MPV, mean platelet volume.

The results of the correlation analysis of AS, SCORE risk calculation, ABI index, and PWVAо with parameters of systemic inflammation and arterial blood gas analysis indicate that the increased fibrinogen concentration is statistically significantly positively correlated with SCORE risk calculation, ABI index, and PWVAо, while hs‐CRP and neutrophil counts are statistically significantly positively correlated with SCORE risk calculation and PWVAо (Table 12).

**Table 12:** Correlation coefficients of arterial stiffness, SCORE risk calculation, ABI index, and PWVAо with inflammatory markers and arterial blood gas analysis parameters

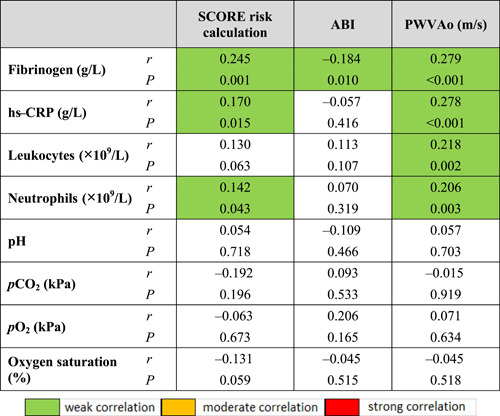

Abbreviations: ABI, ankle‐brachial index; hs‐CRP, high sensitivity protein; PWVAo, aortic pulse wave velocity; *r*, correlation coefficient.

Considering the nonparametric distribution of COPD phenotype and GOLD diagnosis (ABCD tool) (Table 13), a statistically significant positive correlation was found between GOLD COPD diagnosis with SCORE and PWVAo according to the principle of higher GOLD classification. with higher SCORE risk calculation (*r*_s_ = 0.282; *p* = 0.029) and higher PWVAо (*r*_s_ = 0.257; *p* = 0.047).

**Table 13:** Spearman's correlation coefficients of COPD phenotype and COPD assessment according to GOLD in relation to SCORE risk calculation, PWVAо, hs‐CRP, and fibrinogen

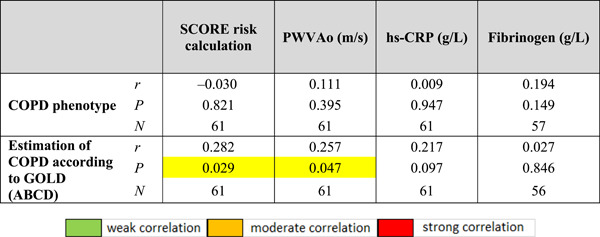

Abbreviations: hs‐CRP, high sensitivity protein; PWVAo, aortic pulse wave velocity; *r*, correlation coefficient.

## 5 DISCUSSION

COPD is currently the only chronic disease showing a significant increase in mortality. In the last 30 years, the mortality rate from COPD has doubled, while at the same time the mortality rate from CV disease has fallen by more than 30%, and a further increase in the incidence of COPD is projected in the next 20 years.^34^ According to the recommendations of the 2017 GOLD initiative, a combined assessment of COPD using ABCD tools is currently being conducted, while the level of obstruction is still ranked according to FEV1 values from zero to four. Level 2 obstruction by GOLD corresponds to moderate and level 3 severe obstructive disturbances. In the sample of this study, the level of obstruction GOLD 2 and 3 prevails within the COPD group, which is 67% of the total number of subjects in the COPD group, similar to the population model of obstruction severity distribution of the Dutch cross‐sectional family physician database where most COPD patients had obstruction level GOLD 2.^35^

By analyzing the clinical characteristics of the group diagnosed with COPD, the most common phenotype of COPD in this study was a nonexacerbator with emphysema (54.1%), while the total percentage of subjects who were nonexacerbators (including nonexacerbators with chronic bronchitis) was 72% in our sample. The reason for such a high share of nonexacerbators can be seen from two aspects. One is that these patients receive adequate therapy in regular pulmonary examinations, given that our sample consists entirely of subjects treated by pulmonologists and we can conclude that there is a good agreement in the distribution of clinical phenotypes with the FENEPOC study in the group of pulmonologically controlled patients.^36^ Another reason is that one of the aims of this study was to detect an increased CV risk in patients with COPD without proven comorbidities that would interfere with the measurements, therefore we did not include patients with comorbidities. Namely, such patients are more prone to exacerbations, which is confirmed by a recent study by Dutch authors which showed that patients with COPD, who have one or more comorbidities, more often have ≥2 exacerbations per year,^37^ therefore based on a sample of patients with COPD in this study cannot draw conclusions about the distribution of the COPD phenotype outside the sample. Also, it is particularly worth noting the fact that this study analyzed data from subjects with COPD without proven comorbidities, which according to population‐based studies in which they make up about 10% of the sample, are the exception rather than the rule.^37^

The analysis of anthropometric parameters (height, body weight, BMI, length of the jugulum symphysis) did not show statistically significant differences, which differs from previous studies in which a negative correlation between BMI and obstruction levels according to GOLD was proven.^38^ This result can be explained by applying the criteria included in this study. Namely, patients with advanced COPD more often have lower BMI and more comorbidities, as well as obese patients, and then BMI contributes to the predictive power of the BODEx index, hence in the population of this study, which consisted of patients with “isolated” COPD without comorbidities, BMI had no predictive value.

Although this study included subjects without a confirmed diagnosis of arterial hypertension, which was the sole criterion, significant differences in systolic and diastolic pressure, heart rate per minute, and mean arterial pressure were noted between the study groups. The mean value of systolic pressure in all groups of the examined population does not exceed the value above 140 mmHg. One‐way analysis of variance showed that the value of systolic pressure was significantly higher in the group of subjects with COPD compared to both control groups, while post hoc analysis confirmed a weak correlation between systolic pressure and GOLD level of obstruction (*r* = 0.264, *p* < 0.001). These results are consistent with the evidence presented by Mannino et al. analyzing data from 20,296 patients in the ARIC (Atherosclerosis Risk in Communities Study) and CHS (Cardiovascular Health Study) studies in which arterial hypertension was found to be one of the most common comorbidities affecting 40%–60% of patients with COPD.^39^ By analyzing the values of diastolic pressure, statistically significant differences were found between the COPD group and the control group of nonsmokers without COPD, while no statistically significant differences were found between the control groups of smokers and nonsmokers. This result can be interpreted by incremental exposure to cigarette smoke, therefore statistical significance is manifested only in the groups with the largest difference in PYI. Namely, in a sample of this study, it was found that respondents with COPD smoke on average twice as many cigarettes as smokers without COPD. Although a recent study described an association between smoking and elevated diastolic blood pressure in elderly subjects, this difference, in contrast to this study, was expressed only in the group of heavy older smokers, while no statistically significant difference was found between groups of moderate or low smokers.^40^

By comparing the values of mean arterial pressure and central systolic aortic pressure between the examined groups, in the COPD group, statistically significantly higher values of these parameters were proved compared to the control groups of smokers and nonsmokers without COPD. Also, additional analysis by Pearson correlation coefficients confirmed a weak correlation between mean arterial pressure and GOLD obstruction level (*r* = 0.250, *p* < 0.001). These results confirm the previously presented research data in which significant differences in blood pressure parameters between the examined groups were investigated.^41^ However, in addition to using a different device in the study, it also included subjects with COPD who had CV comorbidities and did not show whether there was a difference between smokers and nonsmokers without COPD. The data of the second group of researchers did not indicate significant differences in the examined and control group in the values of mean arterial pressure.^26^ There was no control group of nonsmokers in this study and subjects with comorbidities were included. Also, a cross‐sectional national survey with representative data for the entire South Korean population (KNHANES V), conducted between 2010 and 2012, showed that COPD was independently associated with hypertension.^42^

The results of several studies indicate that the level of aortic stiffness in patients with COPD is increased compared to control groups (after correction for age and sex) with positive anamnestic data on smoking.^26^, ^43^, ^44^ Aortic stiffness, measured using PWVAo, is an independent factor in the prediction of CV disease, but it is still not a diagnostic method implemented in everyday practice.^22^, ^24^, ^45^ To date, an association between AS and a reduction in the incidence of CV events has been reported in only one study in a limited sample of patients with advanced renal disease.^46^ Also, in the Framingham study cohort, it has been shown that aortic stiffness further contributes to traditional CV risk factors in predicting the degree of risk.^47^ In this study, the value of PWVAо, as an immediate marker of AS, was also statistically significantly higher in the COPD group compared to the control groups of smokers and nonsmokers without COPD. The mean value of PWVAo in the COPD group (11.67 ± 2.72 m/s, *p* < 0.001) exceeds the critical limit of 10 m/s, which further indicates an increased risk of CV disease and mortality in patients with COPD. The increased value of PWVAо is also a reflection of the significantly shorter P2 pulse wave reflection time in the group of subjects with COPD, which is in line with a recent study by Mills et al.^41^ Linear regression analysis of the significant relationship between PWVAo and GOLD obstruction (*r* = 0.496; *p* < 0.001) showed that as broncho‐obstruction progresses according to the equation *y* = 9.39 + 0.92*x*, AS increases, which clearly indicates that the value of AS will be normal in absence of obstruction, while already at obstruction level 1 this value will exceed the critical point of pathologically elevated PWVAо (>10 m/s). Also, for each degree of obstruction, the PWVAо value increases by 0.92 m/s, which has significant implications for CV risk assessment. The importance of the clinical implications of increased aortic stiffness is increasing for both macrovascular and microvascular disease, as an increase in PWVAo of 1 m/s has been shown to indicate a 15% increase in CV and total mortality.^47^, ^48^ Given the above, it is not surprising that the addition of PWVAo to standard risk factors in the Framingham cohort risk assessment predicts the first CV events and improves the 10‐year risk assessment by 13%.^49^ As already mentioned, PWVAo is still not part of routine everyday clinical practice, although the possibility of including PWVAo in it has been discussed for almost a decade, even at the level of the US Food and Drug Administration.^50^

Numerous studies indicate a two‐way relationship between COPD and the CV disease.^51^, ^52^ In one direction, patients with the coronary artery disease suffering from COPD have twice the risk of the CV disease compared to patients without COPD. In the second direction, patients with COPD have a higher risk of morbidity and mortality than CV disease.^53^, ^54^ In this study, observing the level of obstruction according to GOLD and the value of SCORE risk calculation (Figure 2), a significant correlation was shown that follows the linear function (*p* < 0.001). The higher level of obstruction according to GOLD is significantly associated with poorer results of SCORE calculation of CV risk, and the association follows the regression line *y* = 2.65 + 2.29*x*, that is, GOLD = 1→SCORE = 4.94%; GOLD = 2→SCORE = 7.23%; GOLD = 3→SCORE = 9.52%; GOLD = 4→SCORE = 11.81%. These results suggest that patients with COPD with each degree of obstruction have a higher CV risk by 2.29%. Also, the linear regression correlation found a significant correlation between PWVAо and the CV risk value of a particular SCORE calculation (Figure 1). According to the function of the regression direction, the critical value of PWVAо of 10 m/s is achieved already with the results of SCORE calculation of 4%. These results suggest that the results of AS measurements are significantly related to the SCORE risk calculation.

Hs‐CRP as an indicator of low‐intensity systemic inflammation is now used to grade the general population in CV risk assessment, so some authors report a mild degree of risk at concentrations of 0 to 1 mg/L, a moderate degree of 1 to 3 mg/L, and a high degree risk at concentrations >3 mg/L.^55^, ^56^ Namely, tobacco smoke has been shown to affect the inflammatory process by activating the NF‐κB metabolic pathway and thus stimulate the transcription of genes involved in the innate immune response.^57^, ^58^ Thus, exposure to tobacco smoke leads to a complex systemic inflammatory response through the release of cytokines such as IL‐6 and thus indirectly to enhanced CRP synthesis in the liver.^58^, ^59^ Hs‐CRP has been shown to be a particularly sensitive inflammatory marker and predictor of CV events and may be useful in selecting high‐risk candidates for intensive smoking cessation programs.^60^, ^61^ The earlier presence of systemic inflammation is the most common hypothesis explaining the presence of the CV disease in patients with COPD. Inflammatory markers that have been shown to be elevated in COPD include IL‐6, TNF‐α, and fibrinogen in addition to CRP.^62^ Also, studies indicate that CRP concentration is associated with increased mortality from COPD and that it negatively correlates with FEV1 values.^63^, ^64^ In this study, subjects in the COPD group also had a statistically significantly higher value of hs‐CRP concentration. The COPD group had average hs‐CRP values of 8.81 g/L, the nonsmoking group 3.56 g/L, while the smoking group had an average value of 1.61 g/L. Despite expectations, the nonsmoking group had higher hs‐CRP values than the smoking group, but this difference was not statistically significant. This can be explained by the fact that hs‐CRP is a highly sensitive marker of inflammation and it is possible that in some subjects in the nonsmoking group hs‐CRP was elevated for some other reasons that were not covered by the exclusive criteria or were obvious when taking medical history or physical examination. These results are consistent with the GENOA (The Genetic Epidemiology Network of Arteriopathy study) study, which, among other things, investigated the association between smoking and inflammation. The authors did not find a significant association between smoking intensity and any of the inflammatory markers studied.^65^ Also, in addition to hs‐CRP, in this study, a statistically significantly higher value and concentration of fibrinogen (3.93 ± 1.24 g/L, 95% CI: 3.60–4.26, *p* < 0.001) was found in the COPD group compared to control groups without COPD. These results of higher fibrinogen concentrations are consistent with the results of a meta‐analysis by Manina et al. in which more than half of subjects with COPD had fibrinogen concentration values ≥3.5 g/L, which was associated with an increased risk for future hospitalizations due to acute exacerbation of COPD and increased overall mortality. The authors recommend the use of high fibrinogen values in the screening of high‐risk patients with COPD.^66^ However, shortly after the publication of Manin's meta‐analysis, Faner and Agusti asked why high fibrinogen concentrations in stable disease should be able to predict the risk of severe exacerbations if the CV component of these exacerbations is not taken into account.^67^, ^68^ The authors hypothesized that elevated fibrinogen concentrations reflect its increased pulmonary production. Since the CR3 complement receptor, detected on neutrophils, NK cells, and macrophages, has the ability to bind fibrin, the authors hypothesize this mechanism as a possible option to explain the association between fibrinogen concentration and pneumonia.^69^

The results of the post hoc analysis of correlation coefficients indicate a significant correlation between the concentration of hs‐CRP and fibrinogen with the level of obstruction according to GOLD and all measured spirometric parameters, which confirmed the results of previous studies (Table 10).^63^, ^64^ Also, by post hoc analysis of the results of all smokers in the sample, a significant association between hs‐CRP and fibrinogen with the pack‐year index was found. From the above, we can conclude that twice as much exposure to tobacco smoke is an important factor in the group of subjects with COPD compared to the group of smokers without COPD, therefore the higher the exposure the higher the concentration of hs‐CRP is. The results of this study also indicate a significant positive correlation of hs‐CRP, fibrinogen, and neutrophil values with SCORE risk calculation values and with PWVAо values, which is a surrogate of AS. The results show that subjects with COPD have higher values of systemic inflammation and SCORE risk calculation as well as AS and thus increased CV risk (Table 12). Previous research indicates that fibrinogen is an independent risk factor for CV disease and that hs‐CRP is a significant predictor of future CV events.^60^, ^70^ Stated through a positive correlation with SCORE risk calculation and PWVAо, it further confirms the importance of AS determination in CV risk assessment.

## 6 LIMITATIONS OF THE STUDY

There is a lack of generalizability of findings for age and sex differences since a relatively small clinical study sample (*n* = 208) was analyzed and the sample size calculation was not sufficient to allow the adequate gender stratification of the analyses, but this is a well‐known fact that most COPD subjects are males and over 55 years, as this is accordingly presentable in such a manner in this study.

Comparison analyses were not adjusted for pack‐years in COPD cases when compared with two different control groups of smokers without COPD and nonsmokers without COPD, but this presents no clinical obstacle in obtaining conclusions regarding the fact that in calculations, including CV risk assessment, it is taken in a binary mode.

In this study, patients with comorbidities such as coronary heart disease or atherosclerotic disease of peripheral arteries, arrhythmias or manifest heart failure, unregulated diabetes or hypertension, chronic renal failure, active rheumatic disease, and autoimmune disease since it is recognized that these comorbidities alone affect biological markers of systemic inflammation. However, such a choice introduces a selection bias that renders the study subjects not representative of the universe of the COPD patients, most of whom have comorbidities.

## 7 CONCLUSION

Elevated CV risk was found in subjects with COPD compared to control groups of smokers and nonsmokers without COPD. Subjects with COPD had significantly higher values of SCORE calculation of risk, central aortic pressure, AS, and markers of systemic inflammation (hs‐CRP, fibrinogen). The results of the research support further identification and research of biological markers and affordable and accessible specific tests such as arteriography are promising in providing a better understanding of the complexity and heterogeneity of COPD, advances in personalized treatment, and better primary and secondary prevention of comorbidities for improved treatment outcomes.

## AUTHOR CONTRIBUTIONS

*Conceptualization*: Đivo Ljubičić, Vedran Balta, Dario Dilber. *Formal analysis*: Vedran Balta, Dario Dilber. *Funding acquisition*: Đivo Ljubičić, Dario Dilber. *Writing—review and editing*: Đivo Ljubičić, Vedran Balta, Dario Dilber, Hrvoje Vražić, Domagoj Đikić, Dyana Odeh, Jasna Čerkez Habek, Emilija Lozo Vukovac, Neven Tudorić. *Writing—original draft*: Đivo Ljubičić, Vedran Balta, Dario Dilber. All authors have read and approved the final version of the manuscript, corresponding author had full access to all of the data in this study and takes complete responsibility for the integrity of the data and the accuracy of the data analysis.

## CONFLICTS OF INTEREST

The authors declare no conflicts of interest.

## Data Availability

The authors confirm that the data supporting the findings of this study are available within the article (and/or) its supplementary materials.
